# *In Vivo* Anti-Leukemia, Quantum Chemical Calculations and ADMET Investigations of Some Quaternary and Isothiouronium Surfactants

**DOI:** 10.3390/ph6050634

**Published:** 2013-04-29

**Authors:** Ahmed A. El-Henawy, Manal M. Khowdiary, Abdelfattah B. Badawi, Hussein M. Soliman

**Affiliations:** 1 Chemistry Department, Faculty of Science, Al-Azhar University, Nasr City 11727, Cairo, Egypt; 2 Egyptian Petroleum Research Institute, Applied Surfactant Laboratory, Nasr City 11727, Cairo, Egypt; 3 Chemistry Department, Faculty of Science, Um El Qura University, Makkah 21514, Saudi Arabia; 4 Al Farbabi Collage, Riyadh 11195, Saudi Arabia

**Keywords:** isothiouronium, quaternary salts, leukemia, PM3

## Abstract

Anti-leukemia screening of previously prepared isothiouronium and quaternary salts was performed, and some salts exhibited promising activity as anticancer agents. Quantum chemical calculations were utilized to explore the electronic structure and stability of these compounds. Computational studies have been carried out at the PM3 semiempirical molecular orbitals level, to establish the HOMO-LUMO, IP and ESP mapping of these compounds. The ADMET properties were also studied to gain a clear view of the potential oral bioavailability of these compounds. The surface properties calculated included critical micelle concentration (CMC), maximum surface excess (Γ_max_), minimum surface area (A_min_), free energy of micellization (ΔG^o^_mic_) and adsorption (ΔG^o^_ads_).

## 1. Introduction

**Figure 1:**
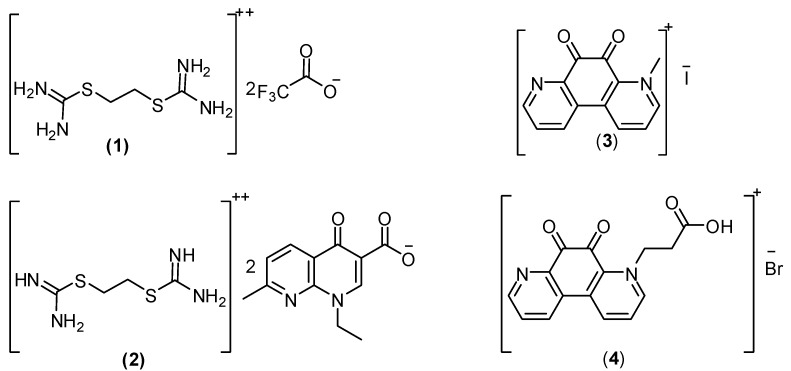
The chemical structure of the previously prepared isothiouronium salts [19].

Cancer is presently considered as one of the primary worldwide health problems, responsible for about 15% of deaths and 25% in developed countries. Antitumor chemotherapy is thus nowaday s a very attractive research target [1,2]. Thioureas, isothiouronium compounds and their derivatives constitute an important class of compounds which exhibit a wide range of antibacterial, fungicidal, herbicidal, antiviral, and plant growth regulatory activities, and play important roles in many chemical and biological processes [3,4,5,6,7], but also act as potential anticancer and anti-HIV drugs [8,9,10,11].The activity of isothiouronium groups may be due to the enhanced acidity of the NH moieties, thereby functioning as a better binder than the thiourea group [12,13,14,15,16,17]. Several synthetic isothiouronium compounds were synthesized to obtain more potent and less toxic therapeutic agents [18].The studies report herein studies aimed to examine the anti-cancer and ADMET properties of some previous synthesized isothiouronium salts **1**–**4** (Figure 1) [19].

## 2. Result and Discussion

### 2.1. Surface Parameter Results for the Prepared Isothornium Salts Surfactants

#### 2.1.1. CMC of the Prepared Surfactants

**Figure 2:**
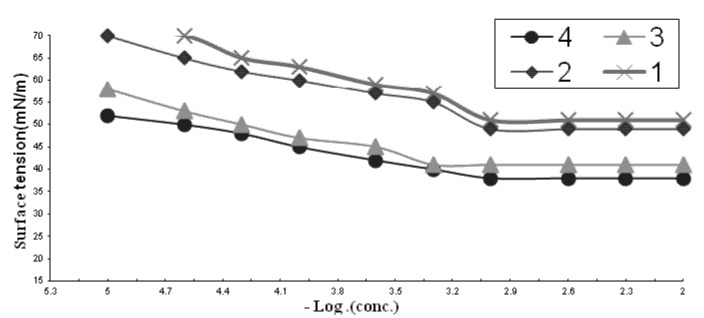
Variation in surface tension of surfactants **1**–**4** *vs*. concentration at 25 °C concentration, expressed as mol/L.

Surfactants form aggregates of molecules or ions called micelles, when the concentration of the surfactant solute in the bulk of the solution exceeds a limiting value, the so called critical micelle concentration (CMC), which is a fundamental characteristic of each solute-solvent system. If the properties of a surfactant solution are plotted as a function of the concentration of the surfactant, the properties usually vary linearly with the concentration, up to critical micelle concentration, at which point there is a break in the curve as shown in Figure 2.

The results in Figure 1 and Table 1 showed that all tested compounds have very low CMC values and a notable decrease of these values was observed in going from **1** to **4**. That fact could be explained from the unique property of the quaternary ammonium salts in water, that is the salts retain their unity in their solutions, which increases their volume in the aqueous media and then repulsion occurs between the hydrophobic chains and water molecules. The lower CMC values of **2** compared with **1** and **4** compared with **3** is due owing to the same reason, whereby an increased hydrophobic part increases this repulsion and accelerates the adsorption of molecules at the interface under very low concentration that form micelles immediately.

**Table 1:** The critical micelle concentration (CMC) and surface parameters of the tested compounds (surfactants).

Comp.	CMC X 10^−3^	γ_CMC_ (mN/m)	Π_CMC_ (mN/m)	P_C20_ (Mole/L)	Γ_max_ X 10^−11^ (Mole/cm^2^)	A _min_ (nm^2^)	Δ G_ads_	Δ G_mic_
**1**	1.3	51	21	3.0	6.9	2.3	−36	−33
**2**	1.05	49	23	3.2	6.5	2.5	−37.5	−33.9
**3**	0.9	41	31	4.5	4.9	3.3	−41	−34.7
**4**	0.6	38	34	5.0	4.5	3.6	−44	−36.4

#### 2.1.2. Effectiveness (Πcmc)

The effectiveness (Πcmc) is determined by the difference between surface tension values at the CMC “γcmc” and the surface tension measured for pure water at the appropriate temperature (γo). The most efficient surfactant one is that gives the greatest lowering in surface tension for a critical micelle concentration (CMC). Compound **4** was found to be the most efficient one in Table 1 because it achieved the maximum reduction of the surface tension at CMC (Figure 2).

#### 2.1.3. Efficiency (PC_20_)

The efficiency (PC_20_) was determined by the concentration (mol/L) capable of suppressing the surface tension by 20 dyne/cm. Values of efficiency of the prepared surfactants are shown in Table 1, the efficiency increased from compound **1** to **4**.

#### 2.1.4. Maximum Surface Excess (Γ_max_)

The number of surfactant molecules at the air-water interface at the critical micelle concentration at (25 °C) is expressed by Γmax. It is evident from Table 1 that Γ_max_ decreases from **1** to **4** this due to the increase in size of the molecules, which are adsorbed at the interface by its huge size and then form the micelles directly at low concentration.

#### 2.1.5. Minimum Area per Molecule (A_min_)

The minimum surface area is defined as the area occupied by a surfactant molecule at the air-water interface at the equilibrium of the solution. Results given in Table 1 indicate that the decrease of Γ_max_ that occurred at the interface was logically accompanied by an increase in A_min_ values from **1** to **4**.

#### 2.1.6. The Standard Free Energies of Micellization (ΔG^o^_mic_) and Adsorption (ΔG^o^_ads_)

From Table 1 the values of ΔG^o^_mic_ and ΔG^o^_ads_ are always negative, indicating the spontaneity of these two processes, although there is more increase in the negativity of ΔG^o^_ads_ than that of micellization, indicating the tendency of the molecules to be adsorbed at the interface.

### 2.2. Anti-Cancer Screening

Tumor growth can be checked by blocking the process of multiplication of tumor cells; this could be achieved either by irradiation or chemical agents. The anticancer activity of the tested compounds **1**–**4** on the life-span of mice bearing Leukemia L-1210 was evaluated (Table 2).

**Table 2:** Anticancer activity of some selected compounds against leukemia L-1210.

Compds.	Dose (µg/kg.)	ILS % *
**1**	400	0
	200	100
	100	100
**2**	400	0
	200	91
	100	99
**3**	400	0
	200	105
	100	120
**4**	400	61
	200	107
	100	114

* Percentage ILS (increase in life span) values were determined from median survival times of treated and saline control groups. If more than 50% of the animals survived for more than 30 days, median survival times and % ILS are indicated as greater than 30 days.

As shown, the isothiuronium compounds **1** and **2** were toxic at a dose level of 400 µg/kg, and failed to increase the life-span of the treated animals. In addition, the quaternary Entobex derivatives **3** and **4**, exhibited borderline activity amounting to 20 and 14%, respectively. From the combination between surface and antitumor activity we reach the conclusion that compounds **3** and **4** with the highest surface activity were also the compounds that exhibited antitumor activity.

In our research surfactants **3** and **4** affected tumor tissues at very low concentrations, lower than their CMC values, which means that there is a strong relation between very small CMC values of these compounds and the display of borderline activity at very low concentration, this being due to the fact that an increasing concentration of surfactant causes an increase in the adsorption process on cell membranes until the CMC is reached, and beyond this concentration the adsorption is slowly retarded and finally stopped due to the formation of micelles which prevent the mobility and suppress anticancer activity.

### 2.3. Molecular Modeling

In trying to gain better insight into the molecular structure of compounds **1**–**4** in Figure 1, conformational analysis of the target compounds has been performed using the MMFF94 force-ﬁeld [20,21] (calculations *in vacuo*, bond dipole option for electrostatics, Polake Ribiere algorithm, RMS gradient of 0.01 kcal/A mol) as implemented in the Spartan 08 program [22]. The most stable conformer was fully geometrically optimized by PM3 [23] semi-empirical *Hamiltonian* molecular orbital calculation [with Restricted Hartree-Fock (RHF) and RMS gradientof 0.05 kca]. Furthermore, the computed molecular parameters, total energy, heat of formation, the lowest occupied molecular orbital (LUMO) and the highest occupied molecular orbital (HOMO) energies, potential energies, solvation energies, electrostatic energies, ionization potential energies, dipole moment and the thermo-dynamic parameters of the studied compounds, such as zero-point vibrational energies, entropy, constant volume molar heat capacity, enthalpy and Gibbs free energy, were calculated (Table 3, Table 4).

**Table 3:** The optimized calculatedenergies and thermodynamic parameters atthe semi-empirical PM3 level for **1**–**4**.

CPD	HF	E	Es	ZPE	S°	C_V_°	H°	G°
**1**	−458.26	−407.84	−43.58	1601.57	462.36	236.63	0.155427819	−0.207933
**2**	−376.38	−446.96	−70.57	628.37	516.22	296.04	0.112537693	0.0539166
**3**	−1835.03	−1914.11	−79.08	514.11	606.16	385.66	−0.436475908	−0.505311
**4**	−508.01	−628.05	−45.80	602.05	857.66	697.30	0.452964763	0.3555698

**HF**: The heat of formation energy (kJ/mol); **E**: Total energy (kJ/mol) (heat of formation + strain energy), **Es**: Solvation energy (kJ/mol), **ZPE**: zero-point vibrational energies (kJ/mol), S°: Entropy (kJ/mol), **C**_v_º: Constant volume molar heat capacity, **H**°: Enthalpy (kJ/mol), **G**°: Gibbs free energy (kJ/mol).

**Table 4:** The optimized calculatedenergies atthe semi-empirical PM3 level for **1**–**4**.

CPD	HOMO	LUMO	HLG	IP	ESP	µ	χ
**1**	−10.21	−2.31	7.9	299.90	77.90	6.04	3.95
**2**	−8.66	−1.32	7.34	51.61	−285.32	5.74	3.67
**3**	−8.03	−1.95	6.08	43.46	−448.58	9.84	3.04
**4**	−8.77	−1.51	7.26	42.94	−279.41	7.97	3.63

**HOMO**: Highest Occupied Molecular Orbital (eV); **LUMO**: Lowest unoccupied Molecular Orbital (eV); **HLG**: difference between HOMO and LUMO energylevels;**IP**: ionization potential energy (kJ/mol); **ESP**: electrostatic potential energy (kJ/mol), **χ**: Mulliken electronegativity (eV), **µ**: dipole moment (Debye).

#### 2.3.1. Energy Features

**Figure 3:**
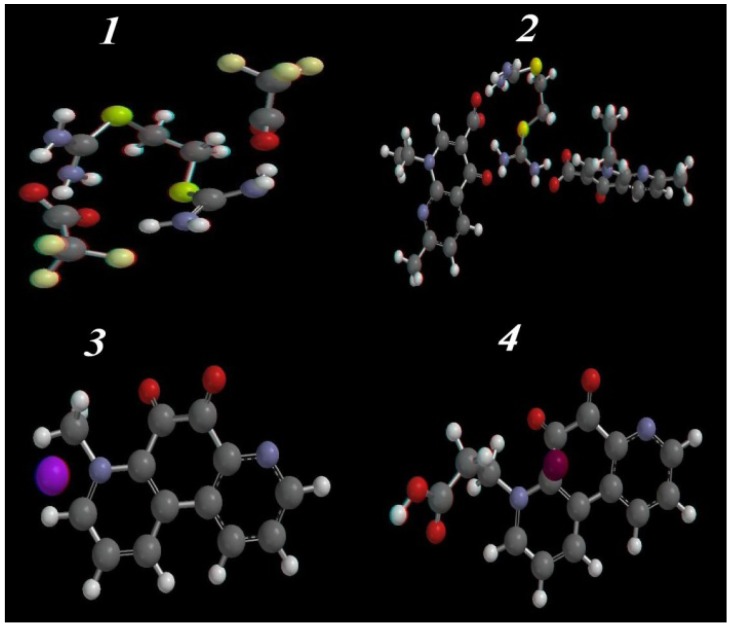
Ball and stick rendering for the most stable conformer of the isothiouronium salts **1**–**4** as calculated by PM3 semi-empirical molecular orbital calculations.

The calculated molecular parameters have been used to investigate the most stable conformer of the compounds **1**–**4** (Table 3), and the optimized geometry structures are represented in Figure 3. The lower calculated energy of the most active compounds **3** and **4** (−1914.11 and −628.05 kJ/mol) compared with the inactiveisothiouronium salts **1** and **2** (−407.84 and −446.96 kJ/mol, respectively), suggests an increase in spatial stability for **3** and **4** compared to **1** and **2**. Furthermore, compound **3** has the lowest calculated solvation energy (−79.08 kJ/mol) which may explain the increasing activity due to increased solubility. Taking into consideration the zero-point energy at 298.15 K temperature, the inter-conversion energy is the largest for **1** and **2**. From comparison of the thermodynamic parameters (Table 4), the most active compounds **3** and **4** are the more stable structures, which agrees with the PM3 calculations.

#### 2.3.2. Optimized Geometry

The single crystal x-ray structures of the isothiouranium compounds **1**–**4** are not available till now, so the geometrical optimization parameters (bond lengths and angles) at thePM3 semi-empirical quantum mechanical level (Figure 3, Figure S1 in Supplementary Information) were calculated (Tables S1–S5 in Supplementary Information).

In **1** and **2**, the N1-C2-N3 and N9-C8-N10 bond angles are ~119°, and the two carbamimidothioate moieties are stabilized by being arranged in a coplanar position with each other, and the distances between C2-N3, N1-C2and C2-S4 are nearly the same as N10-C8, C8-N9 and C8-S7(~1.3, ~1.409 and ~1.8 Å) respectively, and the increased bond length of the imino moiety could be explained by the presence of π electron conjugation of the phenyl ring, and the sp^2^ hybridization N-imino orbitals that has more s-character, with the most electron density closer to the nuclei compared with the electron density distribution at sp^3^ hybridized amino nitrogen, which results in a lengthening of the C_2_-N_1_ and C8-N9 imino bond. The compounds **3** and **4** are arranged in space in the same manner, except the methyl moiety is arranged perpendicular with the phenanthrolinedione ring in **3**, and in **4**, the propionic moiety is arranged coplanar with the phenanthrolinedione ring. The bond lengths in both compounds **3** and **4** for C1-C6, N14-C8, N14-C13 and C9-C10 are about ~1.33, 1.4, 1.31 and 1.500Å, respectively.

#### 2.3.3. HOMO-LUMO and Dipole Moments Analysis

**Figure 4:**
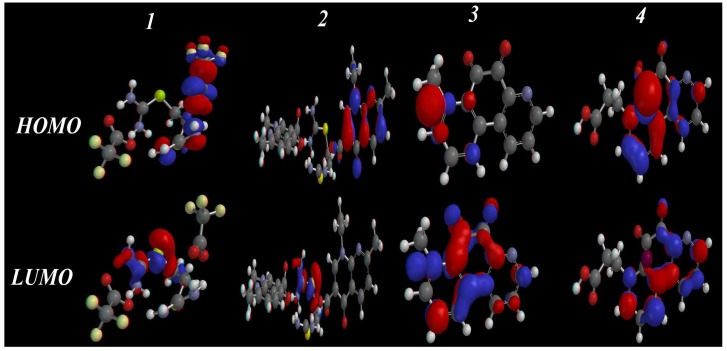
The calculated HOMO, LUMO and HOMO-LUMO gap for **1**–**4** as calculated by the PM3 semiempirical method.

The frontier molecular orbitals (highest occupied molecular orbital, HOMO, and lowest unoccupied molecular orbital, LUMO) are the most important orbitals in a molecule. These orbitals determine the way the molecule interacts with other species. The frontier orbital gap (as calculated by simple Hückel Molecular Orbital theory, SHMO) helps to characterize the chemical reactivity and kinetic stability of amolecule [24,25,26]. Furthermore, the HOMO and LUMO of a molecule play important roles in intermolecular interactions [27], through the interaction between the HOMO of the drug with the LUMO of the receptor and *vice versa*. The interactions are stabilized inversely with the energy gap between the interacting orbitals. Increasing HOMO energy and decreasing LUMO energy in the drug molecule lead to enhancement of stabilizing interactions, and hence, binding with the receptor [27]. HOMOs and LUMOs of the studied systems in the S_0_ states are shown in Figure 4, which suggests the delocalization and localization of molecular orbitals.

The HOMO/LUMO and gap values for compounds **1**–**4** are given in Table 4.The results showed that **3** and **4** have the lowest energy gaps (7.26 and 6.08 eV), and explains their higher affinity towards cancer cells. The total dipole moment is also considered an important physical quantity, as it reflects the interaction ability of the molecules with the surrounding environment. Compounds **3** and **4** have higher dipole moment values (~9.84 and 7.97 Debye) than **1** and **2** (~6.04 and 5.74 Debye), respectively, which increases their ability to interact with the surroundings. Therefore, molecules **3** and **4** are promising structures for interaction with the receptors of cancer cells.

#### 2.3.4. Flexible Alignment

**Figure 5:**
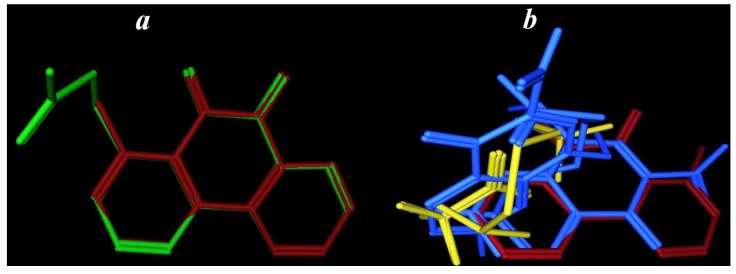
(**a**) Flexible alignments of the most active compounds **3** (in green), **4** and (in red), (**b**) flexible alignment of the highly active compound **3** (in red) and lowest active compounds **1** (in yellow) and **2** (in blue).

To understanding the similarity between the three-dimensional structures of active compounds **3** and **4**, flexible alignment using MOE/MMFF94 was employed (Figure 5) [28].

Two hundred conformers of each compound were generated and minimized with a distance-dependant dielectric model. A low energy set of 100 was selected for further analysis. Conformations of compound **3** were generated using distance geometry and optimized with MMFF94. Thirteen low energy, maximally dissimilar structures were selected for comparison to the other compounds. After assigning MMFF94 charges to all molecules, ﬂexible alignment was ranked by overlays of compounds **3** and **4** based on electrostatic, steric field, hydrophobic areas overlap, hydrogen bond acceptors and donors overlap. From the highest scoring superposition, the limited set of conformers was used in the analysis of molecules with high flexibility capable to achieving complete atom to atom superposition.

A common feature of the MOE-generated alignments was that the two structures showed matched phenanthrolinedione rings, and slight differences in the distance the between methyl and acetyl moieties (0.28Å, Figure 4a). In the same way, the most active anti-cancer compound **3** and the least active compounds **1** and **2** were subjected to flexible alignment. Analysis of the weakly active molecules (Figure 4b), is an important way to gain a clear vision of the essential features for a given activity. It is clear that compounds **1** and **2** were flexibly aligned in a different manner when compared with the active compound **3**. Common feature of the MOE-generated alignments showed, the super position of the dicarbamimidothioate fragments in **1** and **2**, and a deviation distance of about 2.20Å compared with the highly active compound **3**. These features explaining why these compounds were the least active as anti-cancer agents and shows the importance of both the alkyl moiety and the heteroaryl ring attached to it (Figure 5).

#### 2.3.5. Electrostatic (ESP) and Ionization Potential (IP) Map

**Figure 6:**
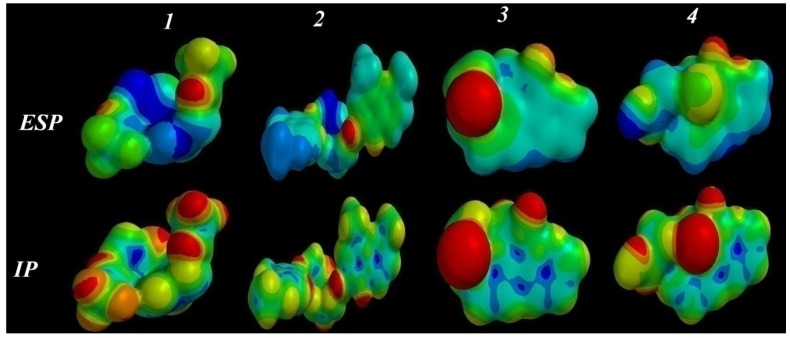
The ESP and IP surfaces for **1**–**4** by PM3 semi-empirical calculations.

In an attempt to understand the lowest and the highest anti-cancer activity of compounds **1**–**4**, electrostatic and ionization potential mapping was performed for the lowest energy conformers, to examine the match and mismatch in electronic, and electrostatic binding characteristics for the molecules surface and conformational properties (Figure 6).

The mapped electrostatic potentials (ESP), represent a balance between the repulsive interactions of the positively-charged nuclei and attractive interactions involving the negatively-charged electrons. The colors toward red depict negative potential (high electron density area, representing a strong attraction between the proton and the points on the molecular surface), while colors toward blue depict positive potential, and colors in between (orange, yellow, green) depict intermediate values of the potential. In addition, the local ionization potential (IP) mapping was performed, which indicates the proportional electron ionization facility around the molecule [29,30] and measure the sensitivity of a molecular zone toward electrophilic attack (reactivity). The default program value used [Fixed Value: (0.032e); Medium Resolution], for the IP surface mapping is shown in Figure 6. Comparison of the ESP and IP mappings of the lowest activity compounds **1** and **2** showed a common feature of increasing distribution of positive charge (in blue), and increase the polar area. On the contrary, the high activity compounds **3** and **4** showed an increased distribution of negative charge (in red) on the surface of the compounds. From the above features, the highest activity may be due to the increasing negative charge located on the compounds, representing hydrogen bond acceptors (in red). It is clear that the related charge distribution and electrostatic ionization mappings suggest a similar interaction and orientations of the molecules with a potential protein-binding site.

### 2.4. ADMET Factor Profiling

Oral bioavailability is considered to play an important role in the development of bioactive molecules as therapeutic agents. Many potential therapeutic agents fail to reach the clinic because of their unfavorable absorption, distribution, metabolism, elimination and toxic (ADMET) factors. Therefore, a computational study for prediction of ADMET properties was performed for compounds **1**–**4**, by the determination of topological polar surface area (TPSA), a calculated percent absorption (%ABS) which was estimated by the Zhao *et al*. equation [31], and the ‘‘rule of five’’, formulated by Lipinski [32],which establish whether a chemical compound can be expected to be an orally active drug in humans, which occurs if it has no more than one violation of the following rules: (i) ClogP (partition coefficient between water and octanol) < 5; (ii) number of hydrogen bond donors sites ≤ 5; (iii) number of hydrogen bond acceptor sites ≤ 10; (iv), molecular weight < 500 and molar refractivity should be between 40–130. In addition, the total polar surface area (TPSA) is another key property linked to drug bioavailability, a the passively absorbed molecules with TPSA > 140 have low oral bioavailability [33]. All calculated descriptors were obtained using the MOE package [32], and the results are listed in Table 5.

**Table 5:** Pharmacokinetic parameters important for good oral bioavailability of compounds **1**–**4**.

CPD	Mwt	TPSA	%ABS	V	LogP	HBD	HBA	Lip-V
1	408.344	187.58	44.2849	255.875	1.58	4	8	0
2	642.76	249.88	22.7914	658.961	2.27	4	14	2
3	352.13	50.88	91.4464	265.068	2.83	0	4	0
4	363.16	88.50	78.4675	307.261	2.035	1	6	0

**TPSA**: Polar surface area; %**ABS**: absorption percentage; **Log P**: Calculated lipophilicity; **LogS**: Solubility parameter; **HBA**: Number of hydrogen bond acceptors; **HBD**: Number of hydrogen bond donors; **Lip-V**: Number of violations of Lipinski’s rule of five; **V**: Volume (Å^3^).

Our results revealed that the CLogP (the lipophilicity factor) [34] was less than 5.0, the molecular weight was less than 500, except for compound **2** (642.76), hydrogen bond acceptor < 10 except for **2** (14) and hydrogen bond donors (~1–4), which fulfill Lipinski’s rule. Also, the percent absorption of compounds **3** and **4** show the highest % absorption among all compounds **1**–**4**. From these data, we can suggest that **3** and **4** can be used as good orally absorbed anti-cancer compounds with less toxicity, but compound **2** is highly toxic and has the lowest oral absorption from all investigated compounds, which matches the empirical data.

### 2.5. Pharmacophore Prediction

**Figure 7:**
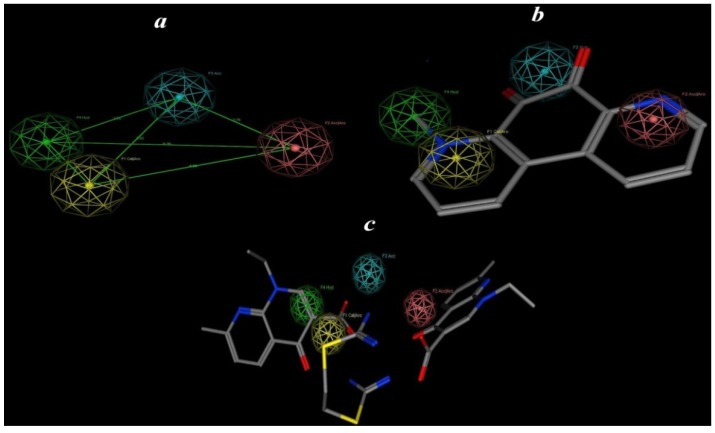
(**a**) Showsthe best predicted pharmacophore features and geometries which are required for anti-cancer activity; (**b**) showsthe most active compound **3** mapped to the pharmacophore model; (**c**) showsthe least active compound **2** mapped to the pharmacophore model. Pharmacophore features are color coded: yellow for catalytic aromatics, green for hydrophobic, red for acceptor aromatics and blue a hydrogen bond acceptor feature.

The aim of this approach is generation and prediction of a pharmacophore model (hypothesis) based on the most active compound. The following steps are employed for 3D pharmacophore applications: (i) Compound **3** is used as reference for other conformations of each compound (Figure 6); (ii) Assignment of the pharmacophoric features; (iii) Application of the method for undertaking conformational searching of databases for new structures matching the generated pharmacophoric features. These steps allow (hypothetical) pharmacophoric building of anticancer compounds. MOE is used for pharmacophore building [28]. The calculated conformational models were performed with a 15 Kcal energy cut off (minimization convergence criteria during conformational analysis: energy convergence = 0.01 kcal/mol, gradient convergence = 0.01 kcal/mol). The number of conformers generated for each substrate was limited to a maximum of 500. All molecules with their associated conformations were regrouped depending on the biological data. Hypothesis generation was carried out with the low energy conformers of the molecules. The calculation and analysis were performed after assignment of possible pharmacophore elements for each analogue using the MOE program, then superposition of the molecules including the assigned elements was attempted (Figure 7), to generate pharmacophore maps, and many runs of the calculations were repeated. 

For each run a distinct number of specified pharmacophore elements were generated. All appropriate models showed that the acceptor atoms of the CO fragments (as acceptor) and the methyl fragment (as hydrophobic unit) were well superimposed within the set distance tolerance, which confirms the important role of the hydrophobic moieties for anticancer activity. The initial pharmacophoric query was carried out, and the introduction of four features, as summarized in Table 6, is illustrated graphically in Figure 7.

**Table 6:** Pharmacophoric and structure features of the most active compounds.

Pharmacophoric features	Structure features
F1: Aro/Cat	Pyridinyl
F2: Aro/Acc	Alkyl pyridinyl
F3: Acc	CO
F4: Hydro	Alkyl

From Figure 7a, models for **3** and its analog **4** possess pharmacophore elements: the heteroaryl ring (as a hydrophobic moiety) attached to an alkyl group which is in plane with the other heteroaryl ring, two heteroaryl rings attached with a CO group (as a hydrogen bond acceptor region, Figure 7b). In contrast, the model for compound **1** and its analog **2** (Figure 7c) showed mismatch with the mapping of the pharmacophore model (Figure 6a), since the alkyl attached with the heteroaryl part of compound **3** plays an important role in activity. According to the pharmacophore generated by MOE [28] the minimal structural requirements for anti-cancer activity consist of an alkyl fragment (hydrophobic region) attached to a heteroaryl ring (aryl catalysis region), carbonyl fragment (H-bonding acceptor region) and another hetroaryl ring (aryl acceptor region). This pharmacophoric assumption was consistent with biological data.

## 3. Experimental

### 3.1. Surface Active Properties Evaluation Methods

#### 3.1.1. Surface Tension Measurements

Surface tension measurement of the prepared surfactants was made at (25 °C) with Du Nouytensiometer (Kruss type 8451) using distilled water solution of 0.1% weight concentration [35]. The surface tension of the used distilled water was 73 mN/m, surfactant solutions were aged for 1/2 h before any measurements were made. Three readings were made on each sample to determine any change with time and to obtain an average value [36].

#### 3.1.2. Efficiency (PC_20_)

The efficiency (PC_20_) was determined as the concentration (mol/L) capable of suppressing the surface tension by 20 dyne/cm [37]. The efficiencies have been determined by extrapolating from γ = 52 to the linear portion before CMC of the γ versus-Logc plot [38], at 25 °C.

#### 3.1.3. Effectiveness (Π_cmc_)

The surface tension (γ_cmc_) values at CMC were used to calculate values of surface pressure (effectiveness) from the following expression:

Π_cmc_ = γ_o_ − γ_cmc_

where γ_o_ is the surface tension measured for the pure water at the appropriate temperature and γ_cmc_ is the surface tension at the CMC. The effectiveness of adsorption is an important factor to determine properties of a surfactant such as foaming, wetting and emulsification, since tightly packed coherent interfacial films have very different interfacial properties than loosely packed, non-coherent films [39].

#### 3.1.4. Determination of Critical Micelle Concentration

CMC of the prepared surfactants were determined by the surface tension method [40]. In this method, values of the surface tension obtained for various concentrations of aqueous solutions of the prepared surfactants were plotted *vs*. the corresponding concentrations.

#### 3.1.5. Maximum Surface Excess Γ_max_

The surface excess concentration is defined as the surface concentration at surface saturation; the maximum surface excess Γ_max_ is a useful measure of the effectiveness of adsorption of the surfactant at the water-air interface, since it is the maximum value at which adsorption can attain:
Γ_max_ =

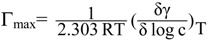

where R = 8.314 J mol^−1^ K^−1^, T is absolute temperature, (δγ/δ logc) is the slope of the surface tension *vs.* log concentration plot at 25 °C [41]. A substance which lowers the surface tension is thus present in excess at or near the surface, *i.e*., when the surface tension decreases with increasing activity of the surfactant, Γ is positive.

#### 3.1.6. Minimum Surface Area (A_min_)

A_min_ is the minimum area per molecule of the prepared compounds at the interface and was calculated from the following equation:
A_min_ =

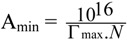

where N is Avogadro’s number and Γ_max_ is the maximum surface excess.

#### 3.1.7. The Standard Free Energies of Micellization ΔG^o^_mic_ and Adsorption ΔG^o^_ads_

Understanding the process of micellization and adsorption are important for explanation of the effects of structural and environmental factors on the value of the CMC and for predicting itseffects on new structural and environmental variations. Standard free energy of micellization ΔG^o^_mic_, and adsorption ΔG^o^_ads_, have played an important role in such understanding.The standard free energy of micellization and adsorption are given by:

ΔG^o^_mic_ = RTln CMC

ΔG^o^_ads_ = ΔG^o^_mic_ − 6.023 × 10^−1^Π_CMC_ A_min_

### 3.2. Anticancer Screening against Leukemia L-1210

Group of six animals were inoculated interperitoneally with 10^5^ cells. After 24 h the tested compounds were injected at three dose levels selected on the basis of their toxicity so that the higher dose is the maximum treatment dose. The animals were kept for one month or till the death of the last animal. The ILS were calculated for all animals died from tumors, and T/C values were calculated.

### 3.3. Molecular Modeling

#### 3.3.1. Computational Details

All electronic structure calculations were performed using the Spartan 08 program [22]. Geometry optimizations have been achieved using the PM3 method. The structural parameters, such as the dipole moment of the molecules µ, the energy of the highest occupied molecular orbital EHOMO and the lowest unoccupied molecular orbital ELUMO were obtained. The thermodynamic parameters, including total energy(TE), enthalpy (H), entropy (S), free energy (G), zero-point vibrational energy (ZPE), the absorption spectra and the vibration frequency calculation For each stationary point was performed at the same levels, to characterize its nature as minima or transition states and to correct energies.

#### 3.3.2. Conformational Search and Flexible Alignment

Conformational analysis and flexible alignment of tested compounds were carried out with the MOE [28] software of Chemical Computing Group Inc. (Montreal, Canada), on a Core 2 duo 3.00 GHz workstation. The molecules were built using the Builder module of MOE. The geometry optimized using the MMFF94 force-field followed by a flexible alignment with systematic conformational search. Lowest energy aligned conformation(s) were identified through the analysis module of DSV.

## 4. Conclusions

Anticancer screening of some previously prepared novel isothiouronium and quaternary salts was performed, and compounds **3** and **4** showed promising activity as anti-leukemia agents. Computational studies have been performed at the PM3 semiempirical molecular orbitals level, to establish the HOMO–LUMO, IP and ESP mapping of these compounds, to understand the lowest and highest anticancer activity of the compounds, and show the importance of the alkyl group in these compounds. Also, the ADMET properties for compounds **3** and **4** showed good oral absorption and suggested they could be used as anti-cancer compounds with less toxicity.

## Supplementary Files

Supplementary File 1 Supplementary Materials (PDF, 315 KB)

## References

[1] Balunas M.J., Kinghorn A.D. (2005). Drug Discovery from medicinal plants. Life Sci..

[2] Parkin D.M. (2001). Global cancer statistics in the year 2000. Lancet Oncol..

[3] Tait A., Gamberini G., Giovannini M.G., di Bella M. (1989). S-aryl (tetramethyl) isothiouronium salts as possible antimicrobial agents, IV. Farmaco.

[4] Tait A., Gamberini G., Giovannini M.G., di Bella M. (1988). S-aryl (tetramethyl) isothiouronium salts as possible antimicrobial agents, III. Farmaco.

[5] Trani A., Ferrari P., Pallanza R., Ciabatti R. (1989). Thioureas and isothiouronium salts of the aglycone of teicoplanin. I. Synthesis and biological activity. J. Antibiot. (Tokyo).

[6] Manez R.M., Sancenon F. (2003). Fluorogenic and chromogenic chemosensors and reagents for anions. Chem. Rev..

[7] Manez R.M., Sancenon F. (2005). New advances in fluorogenic anion chemosensors. J. Fluoresc..

[8] Venkatachalam T.K., Vassilev A.O., Benyunov A., Grigoriants O., Tibbles H.E., Uckun F.M. (2007). Stereochemistry as a Determinant of the Anti-Leukemic Potency of Halopyridyl and Thiazolyl Thiourea Compounds. Lett. Drug Des. Discov..

[9] Manjula S.N., Noolvi N.M., Parihar K.V. (2009). Synthesis and antitumor activity of optically active thiourea and their 2-aminobenzothiazole derivatives: A novel class of anticancer agents. Eur. J. Med. Chem..

[10] Venkatachalam T.K., Mao C., Uckun F.M. (2004). Effect of stereochemistry on the anti-HIV activity of chiral thiourea compounds. Bioorg. Med. Chem..

[11] Hunter R., Kaschula C.H., Parker I.M., Caira M.R., Richards P., Travis S., Taute F., Qwebani T. (2008). Substituted ajoenes as novel anti-cancer agents. Bioorg. Med. Chem. Lett..

[12] Kubo Y., Ishihara S., Tsukahara M., Tokita S. (2002). Isothiouronium-derived simple fluorescent chemosensors of anions. J. Chem. Soc. Perkin Trans. 2.

[13] Kubo Y., Kato M., Misawa Y., Tokita S. (2004). A fluorescence-active 1,3-bis(isothiouronium)-derived naphthalene exhibiting versatile binding modes toward oxoanions in aqueous MeCN solution: New methodology for sensing oxoanions. Tetrahedron Lett..

[14] Kubo Y., Tsukahara M., Ishihara S., Tokita S. (2000). A simple anion chemosensor based on a naphthalene-thiouronium dyad. Chem. Commun..

[15] Nishizawa S., Cui Y.Y., Minagawa M., Morita K., Kato Y., Taniguchi S., Kato R., Teramae N. (2002). Conversion of thioureas to fluorescent isothiouronium-based photoinduced electron transfer sensors for oxoanion sensing. J. Chem. Soc. Perkin Trans. 2.

[16] Yeo W.S., Hong J.I. (1998). Thiouronium-thymine conjugate as a new carrier for selective transport of 5'-AMP. Tetrahedron Lett..

[17] Srivastava S. (2003). Electrodeposition of Copper Metal in Presence of Organic Solvents. Ph.D. Thesis.

[18] Yeo W.S., Hong J.I. (1998). Oxoanion recognition by a thiouronium receptor. Tetrahedron Lett..

[19] Badawi A.M., Seliem V.R., Haroun B., Solima H. (1988). Isothironium salts of potential biological activity. Oriental J.Chem..

[20] Profeta S., Allinger N.L. (1985). Molecular mechanics calculations on aliphatic amines. J. Am. Chem. Soc..

[21] Halgren T.A. (1996). Merck molecular force field I. Basis, form, scope, parameterization, and performance of MMFF94. J. Comput. Chem..

[22] (2008). Spartan 08.

[23] Stewart J.J.P. (1989). MOPAC Manual. J. Comput. Chem..

[24] Rauk A. (2001). Orbital Interaction Theory of Organic Chemistry.

[25] Streitwieser A. (1961). Molecular Orbital Theory for Organic Chemists.

[26] Fleming I. (1976). Frontier Orbitals and Organic Chemical Reactions.

[27] Fukui K. (1982). Role of Frontier Orbitals in Chemical Reactions. Science.

[28] (2009). Computational Chemistry (MOE 2009.10).

[29] Sjoberg P., Murray J.S., Brinck T., Politzer P. (1990). Average local ionization energies on the molecular surfaces of aromatic systems as guides to chemical reactivity. Can. J. Chem..

[30] Politzer P., Murray J.S., Concha M.C. (2002). The complementary roles of molecular surface electrostatic potentials and average local ionization energies with respect to electrophilic processes. Int. J. Quant. Chem..

[31] Zhao Y., Abraham M.H., Lee J., Hersey A., Luscombe N.C., Beck G., Sherborne B., Cooper I. (2002). Rate-limited steps of human oral absorption and QSAR studies. Pharm. Res..

[32] Lipinski C.A., Lombardo F., Dominy B.W., Feeney P.J. (1997). Experimental and computational approaches to estimate solubility and permeability in drug discovery and development settings. Adv. Drug. Delivery Rev..

[33] Clark D.E., Pickett S.D. (2000). Computational methods for the prediction of drug-likeness. Drug Discov. Today.

[34] Wildman S.A., Crippen G.M. (1999). Prediction of Physicochemical Parameters by Atomic Contribution. J. Chem. Inf. Comput. Sci..

[35] Findlay A. (1963). Practical Physical Chemistry.

[36] Hampson J.W., Cosnell D.G. (1996). Surface-tension Properties of Some Poly dispersed Alkyl-Substituted polyoxy-ethylated Phenyl Sulfonamides. J. Am. Oil Chem. Soc..

[37] Takeshita T.I., Shimohara W., Maeda S. (1980). Surface-tension Properties of Some Poly dispersed Alkyl-Substituted. J. Am. Oil Chem. Soc..

[38] Takeshita T.I., Shimohara W., Maeda S. (1982). A Rapid Synthesis of Fatty Acyl Urea Derivatives. J. Am. Oil Chem. Soc..

[39] Bhattacharyya D.N., Kelkar R.Y., Almeida M.R., Das A.K., Chikhale S.V. (1994). Tenside Surfactants Detergents.

[40] Rosen M.J. (1987). Surfactants and Interfacial Phenomena.

[41] Hikota T., Merguro K.J. (1970). Surfactants and interfacial phenomena. Jpn. Chem. Soc..

