# Supplementary material for: In Vivo Anti-Leukemia, Quantum Chemical Calculations and ADMET Investigations of Some Quaternary and Isothiouronium Surfactants
Source: Pharmaceuticals (Basel). 2013 Apr 29;6(5):634–49. doi: 10.3390/ph6050634 (PMC3817726; doi:10.3390/ph6050634)
Supplement: Supplementary File 1 — Supplementary Materials (PDF, 315 KB) [file pharmaceuticals-06-00634-s001.pdf]

Supplementary Materials

Figure S1. Numbering compound **1–4** according to Spartan program.

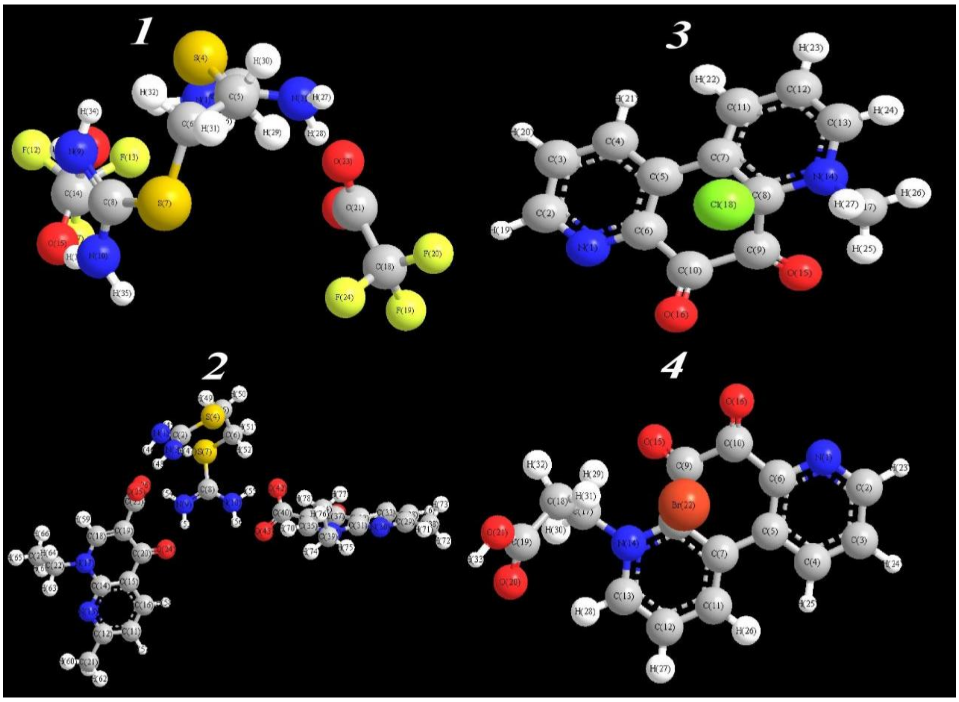

Table S1. Optimized geometrical parameters at Semi-empirical PM3.

**Bond Length Compound 1**	
**Atom NO.**	**Length**
C(8)-N(9)	1.2600
N(1)-H(11)	1.0220
N(9)-H(12)	1.0220
N(3)-H(28)	1.0500
C(6)-H(31)	1.1130
C(6)-H(32)	1.1130
N(1)-C(2)	1.2600
N(10)-H(35)	1.0500
N(10)-H(36)	1.0500
C(8)-N(10)	1.2660
N(3)-H(27)	1.0500
C(8)-S(7)	1.8150
S(7)-C(6)	1.8150
C(6)-C(5)	1.5230
C(5)-S(4)	1.8150
C(2)-S(4)	1.8150
C(2)-N(3)	1.2660
C(5)-H(29)	1.1130
C(5)-H(30)	1.1130

Table S1. *Cont.*

Bond Angle of Compound 1	
**Atom NO.**	**Angle**
H(36)-N(10)-H(35)	120.0000
H(36)-N(10)-C(8)	120.0000
H(35)-N(10)-C(8)	120.0000
H(12)-N(9)-C(8)	120.0000
N(9)-C(8)-N(10)	120.0000
N(10)-C(8)-S(7)	120.0000
C(8)-S(7)-C(6)	120.0000
H(32)-C(6)-H(31)	109.5200
H(32)-C(6)-S(7)	109.4618
H(32)-C(6)-C(5)	109.4618
H(31)-C(6)-S(7)	109.4418
H(31)-C(6)-C(5)	109.4418
S(7)-C(6)-C(5)	109.5000
H(30)-C(5)-H(29)	109.5200
H(30)-C(5)-C(6)	109.4618
H(30)-C(5)-S(4)	109.4618
H(29)-C(5)-C(6)	109.4418
H(29)-C(5)-S(4)	109.4418
C(6)-C(5)-S(4)	109.5000
C(5)-S(4)-C(2)	120.0000
H(28)-N(3)-H(27)	120.0000
H(28)-N(3)-C(2)	120.0000
H(27)-N(3)-C(2)	120.0000
N(1)-C(2)-S(4)	120.0000
N(1)-C(2)-N(3)	120.0000
S(4)-C(2)-N(3)	120.0000
H(11)-N(1)-C(2)	120.0000
**Bond Length Compound 2**	
**Atom NO.**	**Length**
N(1)-H(46)	1.0450
N(9)-H(53)	1.0450
N(9)-H(54)	1.0450
C(5)-H(50)	1.1130
N(10)-H(55)	1.0500
N(3)-H(48)	1.0500
C(6)-H(52)	1.1130
N(10)-H(56)	1.0500
C(6)-H(51)	1.1130
N(3)-H(47)	1.0500
C(5)-H(49)	1.1130
C(8)-N(10)	1.2660
C(8)-N(9)	1.3000
C(8)-S(7)	1.8150
S(7)-C(6)	1.8150
C(6)-C(5)	1.5230
C(5)-S(4)	1.8150
C(2)-S(4)	1.8150
C(2)-N(3)	1.2660
N(1)-C(2)	1.3000
N(1)-H(45)	1.0450
N(10)-H(55)	1.0500

Table S1. *Cont.*

**Bond Length Compound 2**	
**Atom NO.**	**Angle**
H(56)-N(10)-H(55)	120.0000
H(54)-N(9)-H(53)	120.0000
H(54)-N(9)-C(8)	120.0000
H(53)-N(9)-C(8)	120.0000
N(10)-C(8)-N(9)	120.0000
N(10)-C(8)-S(7)	120.0000
N(9)-C(8)-S(7)	120.0000
C(8)-S(7)-C(6)	120.0000
H(52)-C(6)-H(51)	109.5200
H(52)-C(6)-S(7)	109.4618
H(52)-C(6)-C(5)	109.4618
H(51)-C(6)-S(7)	109.4418
H(51)-C(6)-C(5)	109.4418
S(7)-C(6)-C(5)	109.5000
H(50)-C(5)-H(49)	109.5200
H(50)-C(5)-C(6)	109.4618
H(50)-C(5)-S(4)	109.4618
H(49)-C(5)-C(6)	109.4418
H(49)-C(5)-S(4)	109.4418
C(6)-C(5)-S(4)	109.5000
C(5)-S(4)-C(2)	120.0000
H(48)-N(3)-H(47)	120.0000
H(48)-N(3)-C(2)	120.0000
H(47)-N(3)-C(2)	120.0000
S(4)-C(2)-N(3)	120.0000
S(4)-C(2)-N(1)	120.0000
N(3)-C(2)-N(1)	120.0000
H(46)-N(1)-C(2)	120.0000

Table S2. Optimized geometrical parameters at Semi-empirical PM3.

**Bond Angle Compound 3**	
**Atom NO.**	**Length**
C(17)-H(27)	1.1130
C(17)-H(26)	1.1130
C(17)-H(25)	1.1130
C(13)-H(24)	1.1000
C(12)-H(23)	1.1000
C(11)-H(22)	1.1000
C(4)-H(21)	1.1130
C(3)-H(20)	1.1130
N(14)-C(17)	1.5000
C(10)-O(16)	1.2080
C(9)-O(15)	1.2080
C(8)-N(14)	1.3000
C(13)-N(14)	1.3000
C(12)-C(13)	1.2808
C(11)-C(12)	1.3370
C(7)-C(11)	1.3370
C(6)-C(10)	1.3340
C(9)-C(10)	0.9982
C(8)-C(9)	1.3510

Table S2. *Cont.*

C(7)-C(8)	1.3370
C(5)-C(7)	1.3510
N(1)-C(6)	1.2600
C(5)-C(6)	1.3340
C(4)-C(5)	1.3340
C(3)-C(4)	1.3340
C(2)-C(3)	1.3401
N(1)-C(2)	1.2600
C(2)-H(19)	1.1130
**Atom NO.**	**Angle**
H(27)-C(17)-H(26)	108.6875
H(27)-C(17)-H(25)	108.2739
H(27)-C(17)-N(14)	111.7726
H(26)-C(17)-H(25)	108.0021
H(26)-C(17)-N(14)	110.0573
H(25)-C(17)-N(14)	109.9497
C(17)-N(14)-C(8)	116.6403
C(17)-N(14)-C(13)	116.7590
C(8)-N(14)-C(13)	119.3899
H(24)-C(13)-N(14)	116.1095
H(24)-C(13)-C(12)	122.0577
N(14)-C(13)-C(12)	121.7105
H(23)-C(12)-C(13)	120.9198
H(23)-C(12)-C(11)	119.0337
C(13)-C(12)-C(11)	120.0460
H(22)-C(11)-C(12)	118.8720
H(22)-C(11)-C(7)	119.8991
C(12)-C(11)-C(7)	121.2288
O(16)-C(10)-C(6)	125.4733
O(16)-C(10)-C(9)	122.1893
C(6)-C(10)-C(9)	112.3316
O(15)-C(9)-C(10)	122.9536
O(15)-C(9)-C(8)	124.7456
C(10)-C(9)-C(8)	112.2116
N(14)-C(8)-C(9)	114.2709
N(14)-C(8)-C(7)	114.3835
C(9)-C(8)-C(7)	108.9343
C(11)-C(7)-C(8)	120.8081
C(11)-C(7)-C(5)	121.3055
C(8)-C(7)-C(5)	117.8746
C(10)-C(6)-N(1)	117.8675
C(10)-C(6)-C(5)	120.7624
N(1)-C(6)-C(5)	121.3700
C(7)-C(5)-C(6)	121.7371
C(7)-C(5)-C(4)	120.3182
C(6)-C(5)-C(4)	117.9413
H(21)-C(4)-C(5)	119.0083
H(21)-C(4)-C(3)	120.9373
C(5)-C(4)-C(3)	120.0526
H(20)-C(3)-C(4)	120.6453
H(20)-C(3)-C(2)	120.2420
C(4)-C(3)-C(2)	119.1127
C(3)-C(2)-N(1)	121.3102
C(3)-C(2)-H(19)	122.8296
N(1)-C(2)-H(19)	115.8602
C(6)-N(1)-C(2)	120.2124

Table S2. *Cont.*

**Bond Angle Compound 4**	
**Atom NO.**	**Length**
O(21)-H(33)	0.9720
C(18)-H(32)	1.1130
C(18)-H(31)	1.1130
C(17)-H(30)	1.1130
C(17)-H(29)	1.1130
C(13)-H(28)	1.1000
C(12)-H(27)	1.1000
C(11)-H(26)	1.1000
C(4)-H(25)	1.1130
C(3)-H(24)	1.1130
C(19)-O(21)	1.3380
C(19)-O(20)	1.2080
C(18)-C(19)	1.5090
C(17)-C(18)	1.5230
N(14)-C(17)	1.5000
C(10)-O(16)	1.2080
C(9)-O(15)	1.2080
C(8)-N(14)	1.3000
C(13)-N(14)	1.3000
C(12)-C(13)	1.2808
C(11)-C(12)	1.3370
C(7)-C(11)	1.3370
C(6)-C(10)	1.3340
C(9)-C(10)	0.9982
C(8)-C(9)	1.3510
C(7)-C(8)	1.3370
C(5)-C(7)	1.3510
N(1)-C(6)	1.2600
C(5)-C(6)	1.3340
C(4)-C(5)	1.3340
C(3)-C(4)	1.3340
**Atom NO.**	**Angle**
H(33)-O(21)-C(19)	120.0000
O(21)-C(19)-O(20)	120.0000
O(21)-C(19)-C(18)	120.0000
O(20)-C(19)-C(18)	120.0000
H(32)-C(18)-H(31)	109.5200
H(32)-C(18)-C(19)	109.4618
H(32)-C(18)-C(17)	109.4618
H(31)-C(18)-C(19)	109.4418
H(31)-C(18)-C(17)	109.4418
C(19)-C(18)-C(17)	109.5000
H(30)-C(17)-H(29)	109.5200
H(30)-C(17)-C(18)	109.4618
H(30)-C(17)-N(14)	109.4618
H(29)-C(17)-C(18)	109.4418
H(29)-C(17)-N(14)	109.4418
C(18)-C(17)-N(14)	109.5000
C(17)-N(14)-C(8)	122.5000
C(17)-N(14)-C(13)	122.5000
C(8)-N(14)-C(13)	115.0000
H(28)-C(13)-N(14)	115.2101
H(28)-C(13)-C(12)	115.2101
N(14)-C(13)-C(12)	129.5797
H(27)-C(12)-C(13)	123.9736

Table S2. *Cont.*

**Bond Angle Compound 4**	
**Atom NO.**	**Angle**
H(27)-C(12)-C(11)	123.9736
C(13)-C(12)-C(11)	112.0528
H(26)-C(11)-C(12)	120.0000
H(26)-C(11)-C(7)	120.0000
C(12)-C(11)-C(7)	120.0000
O(16)-C(10)-C(6)	117.7209
O(16)-C(10)-C(9)	117.7209
C(6)-C(10)-C(9)	124.5581
O(15)-C(9)-C(10)	117.3837
C(10)-C(9)-C(8)	125.2326
N(14)-C(8)-C(9)	122.3986
N(14)-C(8)-C(7)	119.9987
C(9)-C(8)-C(7)	117.6000
C(11)-C(7)-C(8)	119.9987
C(11)-C(7)-C(5)	122.3986
C(8)-C(7)-C(5)	117.6000
C(10)-C(6)-N(1)	119.9988
C(10)-C(6)-C(5)	119.9988
N(1)-C(6)-C(5)	120.0000
C(7)-C(5)-C(6)	115.0000
C(7)-C(5)-C(4)	124.9984
C(6)-C(5)-C(4)	119.9986
H(25)-C(4)-C(5)	120.0000
H(25)-C(4)-C(3)	120.0000
C(5)-C(4)-C(3)	120.0000
H(24)-C(3)-C(4)	122.7435
H(24)-C(3)-C(2)	122.7435
C(4)-C(3)-C(2)	114.5130
C(3)-C(2)-N(1)	125.4884
C(3)-C(2)-H(23)	117.2558
N(1)-C(2)-H(23)	117.2558
C(6)-N(1)-C(2)	120.0000
C(10)-C(9)-C(8)	125.2326
N(14)-C(8)-C(9)	122.3986
N(14)-C(8)-C(7)	119.9987
C(9)-C(8)-C(7)	117.6000
C(11)-C(7)-C(8)	119.9987
C(11)-C(7)-C(5)	122.3986
C(8)-C(7)-C(5)	117.6000
C(10)-C(6)-N(1)	119.9988
C(10)-C(6)-C(5)	119.9988

Table 3: Optimized geometrical parameters at Semi-empirical PM3.

**Bond Angle Compound 3**		**Atom NO.**	**Angle**	**Atom NO.**	**Length**	**Atom NO.**	**Angle**
**Atom NO.**	**Angle**	C(10)-C(6)-C(5)	120.7624	C(10)-O(16)	0.8824	H(30)-C(17)-C(18)	110.8188
H(27)-C(17)-H(26)	108.6875	N(1)-C(6)-C(5)	121.3701	C(9)-O(15)	0.8868	H(30)-C(17)-N(14)	108.5227
H(27)-C(17)-H(25)	108.2739	C(7)-C(5)-C(6)	121.7371	C(8)-N(14)	1.0393	H(29)-C(17)-C(18)	109.7412
H(27)-C(17)-N(14)	111.7726	C(7)-C(5)-C(4)	120.3182	C(13)-N(14)	1.0224	H(29)-C(17)-N(14)	106.9103
H(26)-C(17)-H(25)	108.0021	C(6)-C(5)-C(4)	117.9414	C(12)-C(13)	0.9964	C(18)-C(17)-N(14)	114.1067
H(26)-C(17)-N(14)	110.0573	H(21)-C(4)-C(5)	119.0084	C(11)-C(12)	1.0372	C(17)-N(14)-C(8)	121.0768
H(25)-C(17)-N(14)	109.9497	H(21)-C(4)-C(3)	120.9372	C(7)-C(11)	1.0002	C(17)-N(14)-C(13)	118.4438
C(17)-N(14)-C(8)	116.6404	C(5)-C(4)-C(3)	120.0526	C(6)-C(10)	1.0915	C(8)-N(14)-C(13)	120.4637
C(17)-N(14)-C(13)	116.7589	H(20)-C(3)-C(4)	120.6454	C(9)-C(10)	1.1163	H(28)-C(13)-N(14)	116.8745
C(8)-N(14)-C(13)	119.3900	H(20)-C(3)-C(2)	120.2420	C(8)-C(9)	1.1111	H(28)-C(13)-C(12)	122.0970
H(24)-C(13)-N(14)	116.1097	C(4)-C(3)-C(2)	119.1126	C(7)-C(8)	1.0679	N(14)-C(13)-C(12)	121.0267
H(24)-C(13)-C(12)	122.0576	C(3)-C(2)-N(1)	121.3102	C(5)-C(7)	1.0694	H(27)-C(12)-C(13)	120.4104
N(14)-C(13)-C(12)	121.7104	C(3)-C(2)-H(19)	122.8295	N(1)-C(6)	0.9925	H(27)-C(12)-C(11)	119.5763
H(23)-C(12)-C(13)	120.9199	N(1)-C(2)-H(19)	115.8602	C(5)-C(6)	1.0287	C(13)-C(12)-C(11)	120.0062
H(23)-C(12)-C(11)	119.0337	C(6)-N(1)-C(2)	120.2123	C(3)-C(4)	1.0137	H(26)-C(11)-C(12)	119.2355
C(13)-C(12)-C(11)	120.0460	**Bond Length Compound 4**		C(2)-C(3)	1.0217	H(26)-C(11)-C(7)	119.9201
H(22)-C(11)-C(12)	118.8720	**Atom NO.**	**Length**	N(1)-C(2)	0.9850	C(12)-C(11)-C(7)	120.8388
H(22)-C(11)-C(7)	119.8991	O(21)-H(33)	0.6962	C(2)-H(23)	0.8016	O(16)-C(10)-C(6)	125.4064
C(12)-C(11)-C(7)	121.2287	C(18)-H(32)	0.8117	C(3)-C(4)	1.0137	O(16)-C(10)-C(9)	121.9937
O(16)-C(10)-C(6)	125.4731	C(18)-H(31)	0.8146	C(2)-C(3)	1.0217	C(6)-C(10)-C(9)	112.5716
O(16)-C(10)-C(9)	122.1894	C(17)-H(30)	0.8114	**Bond Angle Compound 4**		O(15)-C(9)-C(10)	123.5655
C(6)-C(10)-C(9)	112.3317	C(17)-H(29)	0.8214	**Atom NO.**	**Angle**	O(15)-C(9)-C(8)	123.1647
O(15)-C(9)-C(10)	122.9536	C(13)-H(28)	0.8057	H(33)-O(21)-C(19)	110.0108	C(10)-C(9)-C(8)	113.2683
O(15)-C(9)-C(8)	124.7456	C(12)-H(27)	0.8002	O(21)-C(19)-O(20)	115.5647	N(14)-C(8)-C(9)	119.8678
C(10)-C(9)-C(8)	112.2116	C(11)-H(26)	0.8053	O(21)-C(19)-C(18)	114.5818	N(14)-C(8)-C(7)	116.8189
N(14)-C(8)-C(9)	114.2709	C(4)-H(25)	0.8048	O(20)-C(19)-C(18)	129.8508	C(9)-C(8)-C(7)	114.7215
N(14)-C(8)-C(7)	114.3835	C(3)-H(24)	0.8003	H(32)-C(18)-H(31)	105.2707	C(11)-C(7)-C(8)	119.8925
C(9)-C(8)-C(7)	108.9344	C(19)-O(21)	0.9880	H(32)-C(18)-C(19)	108.0297	C(11)-C(7)-C(5)	121.0527
C(11)-C(7)-C(8)	120.8082	C(19)-O(20)	0.8923	H(32)-C(18)-C(17)	108.5042	C(8)-C(7)-C(5)	119.0292
C(11)-C(7)-C(5)	121.3054	C(18)-C(19)	1.1003	H(31)-C(18)-C(19)	109.0665	C(10)-C(6)-N(1)	117.6133
C(8)-C(7)-C(5)	117.8746	C(17)-C(18)	1.1146	C(19)-C(18)-C(17)	114.0063	C(10)-C(6)-C(5)	120.9745
C(10)-C(6)-N(1)	117.8675	N(14)-C(17)	1.0907	H(30)-C(17)-H(29)	106.3883	N(1)-C(6)-C(5)	121.4119

Table S3. *Cont.*

Dihedral Angle compound 1		Dihedral Angle compound 2		Dihedral Angle Compound 3			
**Atom NO.**	**Angle**	**Atom NO.**	**Angle**	**Atom NO.**	**Length**	**Atom NO.**	**Angle**
S(7)-C(8)-N(9)-H(12)	-180.0000	S(7)-C(8)-N(10)-H(55)	15.4935	C(13)-N(14)-C(17)-H(25)	0.0000	H(22)-C(11)-C(12)-H(23)	-19.4628
N(10)-C(8)-N(9)-H(12)	0.0000	S(7)-C(8)-N(10)-H(56)	162.1500	C(13)-N(14)-C(17)-H(26)	119.9636	C(5)-C(7)-C(11)-C(12)	-170.1146
H(11)-N(1)-C(2)-N(3)	180.0000	N(9)-C(8)-N(10)-H(55)	-175.0315	C(13)-N(14)-C(17)-H(27)	-120.0004	C(5)-C(7)-C(11)-H(22)	9.8854
H(11)-N(1)-C(2)-S(4)	0.0000	N(9)-C(8)-N(10)-H(56)	-28.3750	C(8)-N(14)-C(17)-H(25)	-180.0000	C(8)-C(7)-C(11)-C(12)	10.5008
N(9)-C(8)-N(10)-H(35)	0.0000	S(7)-C(8)-N(9)-H(53)	-168.9361	C(8)-N(14)-C(17)-H(26)	-60.0364	C(8)-C(7)-C(11)-H(22)	-169.4992
N(9)-C(8)-N(10)-H(36)	180.0000	S(7)-C(8)-N(9)-H(54)	-25.6468	C(8)-N(14)-C(17)-H(27)	59.9996	C(5)-C(6)-C(10)-C(9)	1.1644
N(9)-C(8)-S(7)-C(6)	-180.0000	N(10)-C(8)-N(9)-H(53)	21.1393	C(7)-C(8)-N(14)-C(13)	0.0000	C(5)-C(6)-C(10)-O(16)	-178.8356
N(1)-C(2)-S(4)-C(5)	180.0000	N(10)-C(8)-N(9)-H(54)	164.4286	C(7)-C(8)-N(14)-C(17)	-180.0000	N(1)-C(6)-C(10)-C(9)	-179.4085
N(1)-C(2)-N(3)-H(27)	180.0000	N(9)-C(8)-S(7)-C(6)	143.6087	C(9)-C(8)-N(14)-C(13)	-179.3846	N(1)-C(6)-C(10)-O(16)	0.5915
N(1)-C(2)-N(3)-H(28)	0.0000	N(10)-C(8)-S(7)-C(6)	-46.6352	C(9)-C(8)-N(14)-C(17)	0.6154	C(8)-C(9)-C(10)-C(6)	-0.4563
S(7)-C(8)-N(9)-H(12)	-180.0000	C(8)-S(7)-C(6)-C(5)	-128.8875	C(12)-C(13)-N(14)-C(8)	-12.3429	C(8)-C(9)-C(10)-O(16)	179.5437
N(10)-C(8)-N(9)-H(12)	0.0000	C(8)-S(7)-C(6)-H(51)	112.0360	C(12)-C(13)-N(14)-C(17)	167.6571	O(15)-C(9)-C(10)-C(6)	179.5437
H(11)-N(1)-C(2)-N(3)	180.0000	C(8)-S(7)-C(6)-H(52)	-2.6672	H(24)-C(13)-N(14)-C(8)	167.6571	O(15)-C(9)-C(10)-O(16)	-0.4563
H(11)-N(1)-C(2)-S(4)	0.0000	S(7)-C(6)-C(5)-S(4)	93.1725	H(24)-C(13)-N(14)-C(17)	-12.3429	C(7)-C(8)-C(9)-C(10)	-0.1282
N(9)-C(8)-N(10)-H(35)	0.0000	S(7)-C(6)-C(5)-H(49)	-36.5051	C(11)-C(12)-C(13)-N(14)	21.8423	C(7)-C(8)-C(9)-O(15)	179.8718
N(9)-C(8)-N(10)-H(36)	180.0000	S(7)-C(6)-C(5)-H(50)	-152.8072	C(11)-C(12)-C(13)-H(24)	-158.1577	N(14)-C(8)-C(9)-C(10)	179.2704
N(9)-C(8)-S(7)-C(6)	-180.0000	H(51)-C(6)-C(5)-S(4)	-149.1217	H(23)-C(12)-C(13)-N(14)	-158.1577	N(14)-C(8)-C(9)-O(15)	-0.7296
N(1)-C(2)-S(4)-C(5)	180.0000	H(51)-C(6)-C(5)-H(49)	81.2008	H(23)-C(12)-C(13)-H(24)	21.8423	C(5)-C(7)-C(8)-C(9)	0.0000
N(1)-C(2)-N(3)-H(27)	180.0000	H(51)-C(6)-C(5)-H(50)	-35.1013	C(7)-C(11)-C(12)-C(13)	-19.4628	C(5)-C(7)-C(8)-N(14)	-179.4137
		H(52)-C(6)-C(5)-S(4)	-33.9025	C(7)-C(11)-C(12)-H(23)	160.5372	C(11)-C(7)-C(8)-C(9)	179.4137
		H(52)-C(6)-C(5)-H(49)	-163.5801	H(22)-C(11)-C(12)-C(13)	160.5372	C(11)-C(7)-C(8)-N(14)	0.0000
		H(52)-C(6)-C(5)-H(50)	80.1178	H(22)-C(11)-C(12)-H(23)	-19.4628	C(4)-C(5)-C(7)-C(8)	180.0000
		C(6)-C(5)-S(4)-C(2)	-74.9738	C(5)-C(7)-C(11)-C(12)	-170.1146	C(4)-C(5)-C(7)-C(11)	0.6014
		H(49)-C(5)-S(4)-C(2)	54.3888	C(5)-C(7)-C(11)-H(22)	9.8854	C(6)-C(5)-C(7)-C(8)	0.6339
		H(50)-C(5)-S(4)-C(2)	166.6753	C(8)-C(7)-C(11)-C(12)	10.5008	C(6)-C(5)-C(7)-C(11)	-178.7648
		N(1)-C(2)-S(4)-C(5)	-26.5328	C(8)-C(7)-C(11)-H(22)	-169.4992	C(2)-N(1)-C(6)-C(5)	0.0000
		N(3)-C(2)-S(4)-C(5)	160.8413	C(5)-C(6)-C(10)-C(9)	1.1644	C(2)-N(1)-C(6)-C(10)	-179.4271
		N(1)-C(2)-N(3)-H(47)	-179.5590	C(5)-C(6)-C(10)-O(16)	-178.8356	C(4)-C(5)-C(6)-N(1)	0.0000
		N(1)-C(2)-N(3)-H(48)	19.5150	N(1)-C(6)-C(10)-C(9)	-179.4085	C(4)-C(5)-C(6)-C(10)	179.4271

Table S3. *Cont.*

**Dihedral bond Angle Compound 3**		**Dihedral bond Angle Compound 4**			
**Atom NO.**	**Angle**	**Atom NO.**	**Length**	**Atom NO.**	**Angle**
C(4)-C(5)-C(6)-C(10)	179.4271	C(18)-C(19)-O(21)-H(33)	-179.5907	H(28)-C(13)-N(14)-C(17)	-6.5402
C(7)-C(5)-C(6)-N(1)	179.4004	O(20)-C(19)-O(21)-H(33)	0.9584	C(11)-C(12)-C(13)-N(14)	-1.6490
C(7)-C(5)-C(6)-C(10)	-1.1725	C(17)-C(18)-C(19)-O(20)	2.3757	C(11)-C(12)-C(13)-H(28)	177.8604
C(3)-C(4)-C(5)-C(6)	0.0000	C(17)-C(18)-C(19)-O(21)	-176.9791	H(27)-C(12)-C(13)-N(14)	179.3246
C(3)-C(4)-C(5)-C(7)	-179.3366	H(31)-C(18)-C(19)-O(20)	-123.0124	H(27)-C(12)-C(13)-H(28)	-1.1660
H(21)-C(4)-C(5)-C(6)	-180.0000	H(31)-C(18)-C(19)-O(21)	57.6328	C(7)-C(11)-C(12)-C(13)	2.6349
H(21)-C(4)-C(5)-C(7)	0.6634	H(32)-C(18)-C(19)-O(20)	123.0615	C(7)-C(11)-C(12)-H(27)	-178.3306
C(2)-C(3)-C(4)-C(5)	0.0000	H(32)-C(18)-C(19)-O(21)	-56.2932	H(26)-C(11)-C(12)-C(13)	-176.5004
C(2)-C(3)-C(4)-H(21)	-180.0000	N(14)-C(17)-C(18)-C(19)	-86.5880	H(26)-C(11)-C(12)-H(27)	2.5341
H(20)-C(3)-C(4)-C(5)	180.0000	N(14)-C(17)-C(18)-H(31)	37.4816	C(5)-C(7)-C(11)-C(12)	-178.3764
H(20)-C(3)-C(4)-H(21)	0.0000	N(14)-C(17)-C(18)-H(32)	152.9915	C(5)-C(7)-C(11)-H(26)	0.7530
H(19)-C(2)-C(3)-C(4)	180.0000	H(29)-C(17)-C(18)-C(19)	153.4688	C(8)-C(7)-C(11)-C(12)	3.4831
H(19)-C(2)-C(3)-H(20)	0.0000	H(29)-C(17)-C(18)-H(31)	-82.4616	C(8)-C(7)-C(11)-H(26)	-177.3875
N(1)-C(2)-C(3)-C(4)	0.0000	H(29)-C(17)-C(18)-H(32)	33.0483	C(5)-C(6)-C(10)-C(9)	-23.5325
N(1)-C(2)-C(3)-H(20)	180.0000	H(30)-C(17)-C(18)-C(19)	36.2651	C(5)-C(6)-C(10)-O(16)	158.3764
C(6)-N(1)-C(2)-H(19)	180.0000	H(30)-C(17)-C(18)-H(31)	160.3347	N(1)-C(6)-C(10)-C(9)	156.6744
C(6)-N(1)-C(2)-C(3)	0.0000	H(30)-C(17)-C(18)-H(32)	-84.1554	N(1)-C(6)-C(10)-O(16)	-21.4166
C(4)-C(5)-C(6)-C(10)	179.4271	C(13)-N(14)-C(17)-C(18)	89.3960	C(8)-C(9)-C(10)-C(6)	45.8336
C(7)-C(5)-C(6)-N(1)	179.4004	C(13)-N(14)-C(17)-H(29)	-149.0834	C(8)-C(9)-C(10)-O(16)	-136.0009
C(7)-C(5)-C(6)-C(10)	-1.1725	C(13)-N(14)-C(17)-H(30)	-34.6985	O(15)-C(9)-C(10)-C(6)	-133.7282
C(3)-C(4)-C(5)-C(6)	0.0000	C(8)-N(14)-C(17)-C(18)	-92.0428	O(15)-C(9)-C(10)-O(16)	44.4373
C(3)-C(4)-C(5)-C(7)	-179.3366	C(8)-N(14)-C(17)-H(29)	29.4778	C(7)-C(8)-C(9)-C(10)	-46.6721
		C(8)-N(14)-C(17)-H(30)	143.8627	C(7)-C(8)-C(9)-O(15)	132.8917
		C(7)-C(8)-N(14)-C(13)	11.2453	N(14)-C(8)-C(9)-C(10)	166.4854
		C(7)-C(8)-N(14)-C(17)	-167.2869	N(14)-C(8)-C(9)-O(15)	-13.9508
		C(9)-C(8)-N(14)-C(13)	157.4177	C(5)-C(7)-C(8)-C(9)	23.7117
		C(9)-C(8)-N(14)-C(17)	-21.1145	C(5)-C(7)-C(8)-N(14)	171.6075
		C(12)-C(13)-N(14)-C(8)	-5.5765	C(11)-C(7)-C(8)-C(9)	-158.1101
		C(12)-C(13)-N(14)-C(17)	172.9938	C(11)-C(7)-C(8)-N(14)	-10.2144
		H(28)-C(13)-N(14)-C(8)	174.8894	H(28)-C(13)-N(14)-C(17)	-6.5402

Table 4. Mulliken q(M) and neutral charges q(n) for **1, 2, 3** and **4** at Semi-empirical PM3.

Atom No.	q(M)	q(n)
**1 and 2**		
N0 :	-0.529	+0.141
C1 :	+0.086	-0.306
N2 :	-0.508	+0.151
S3 :	-0.266	-0.071
C4 :	-0.010	-0.204
C5 :	-0.010	-0.204
S6 :	-0.266	-0.071
C7 :	+0.086	-0.306
N8 :	-0.529	+0.141
N9 :	-0.508	+0.151
H1 :	+0.274	+0.059
H2 :	+0.313	+0.014
H3 :	+0.277	+0.066
H4 :	+0.302	+0.013
H5 :	+0.076	+0.061
H6 :	-0.014	+0.076
H7 :	-0.014	+0.076
H8 :	+0.076	+0.061
H9 :	+0.313	+0.014
H10 :	+0.274	+0.059
H11 :	+0.277	+0.066
H12 :	+0.302	+0.013
**3**		
C1 :	-0.074	-0.074
C2 :	-0.113	-0.113
N1 :	-0.002	-0.002
C3 :	-0.085	-0.085
C4 :	-0.011	-0.011
C5 :	-0.111	-0.111
C6 :	-0.118	-0.118
C7 :	+0.031	+0.031
C8 :	+0.258	+0.258
C9 :	+0.314	+0.314
C10 :	+0.003	+0.003
C11 :	-0.188	-0.188
C12 :	-0.108	-0.108
N13 :	+0.236	+0.236
O14 :	-0.267	-0.267
O15 :	-0.212	-0.212
C16 :	-0.136	-0.136
C17 :	-0.130	-0.130
C18 :	+0.388	+0.388
O19 :	-0.419	-0.419
O20 :	-0.295	-0.295
H1 :	+0.126	+0.126
H2 :	+0.117	+0.117
H3 :	+0.117	+0.117
H5 :	+0.130	+0.130
H7 :	+0.130	+0.130
H8 :	+0.091	+0.091
H9 :	+0.128	+0.128
H10 :	+0.098	+0.098
H11 :	+0.230	+0.230
**4**		
C1 :	+0.461	-0.074
C2 :	-0.432	-0.113
C3 :	+0.252	-0.085
C4 :	-0.372	-0.011
C5 :	+0.486	-0.111
C6 :	-0.063	-0.118
C7 :	-0.080	+0.031
C8 :	+0.558	+0.258
C9 :	+0.206	+0.314
C10 :	+0.021	+0.003
C11 :	-0.249	-0.188
C12 :	-0.116	-0.108
N13 :	+0.413	+0.236
O14 :	-0.444	-0.267
H1 :	+0.027	+0.126
H2 :	+0.145	+0.117
H3 :	+0.061	+0.117
H4 :	+0.121	+0.118
H5 :	+0.140	+0.130
H6 :	+0.177	+0.160
H7 :	+0.242	+0.130
H8 :	+0.187	+0.091
H9 :	+0.154	+0.128
H10 :	+0.166	+0.098
H11 :	+0.417	+0.230
